# Influence of Preparation Temperature on the Properties and Performance of Composite PVDF-TiO_2_ Membranes

**DOI:** 10.3390/membranes11110876

**Published:** 2021-11-15

**Authors:** Duc-Trung Tran, Jean-Pierre Méricq, Julie Mendret, Stephan Brosillon, Catherine Faur

**Affiliations:** IEM (Institut Européen des Membranes), UMR 5635 (CNRS-ENSCM-UM2), Université de Montpellier, 34095 Montpellier, France; ductrungtr@gmail.com (D.-T.T.); jean-pierre.mericq@umontpellier.fr (J.-P.M.); stephan.brosillon@umontpellier.fr (S.B.); catherine.faur@umontpellier.fr (C.F.)

**Keywords:** PVDF, TiO_2_, photocatalytic membrane, temperature, non-solvent-induced phase separation (NIPS)

## Abstract

Composite PVDF-TiO_2_ membranes are studied extensively in literature as effective anti-fouling membranes with photocatalytic properties. Yet, a full understanding of how preparation parameters affect the final membrane structure, properties and performance has not been realized. In this study, PVDF-TiO_2_ membranes (20 wt% TiO_2_/PVDF) were fabricated via the non-solvent-induced phase separation (NIPS) method with an emphasis on the preparation temperature. Then, a systematic approach was employed to study the evolution of the membrane formation process and membrane properties when the preparation temperature changed, as well as to establish a link between them. Typical asymmetric membranes with a high porosity were obtained, along with a vast improvement in the permeate flux compared to the neat PVDF membranes, but a reduction in mechanical strength was also observed. Interestingly, upon the increase in preparation temperature, a significant transition in membrane morphology was observed, notably the gradual diminution of the finger-like macrovoids. Other membrane properties such as permeability, porosity, thermal and mechanical properties, and compression behavior were also influenced accordingly. Together, the establishment of the ternary phase diagrams, the study of solvent–nonsolvent exchange rate, and the direct microscopic observation of membrane formation during phase separation, helped explain such evolution in membrane properties.

## 1. Introduction

Compared to conventional water treatment technologies, membrane separation processes are highly efficient in the removal of particles, microorganisms, or even dissolved organic matters, yet consume no chemicals, or very small amounts of chemicals, for cleaning. However, membrane fouling has always been a critical problem in microfiltration (MF) and ultrafiltration (UF) processes, causing a significant decline in filtration performance over time, which leads to high energy consumption, environment-related costs and, eventually, the replacement of the membranes. A straightforward way to deal with this problem is to enhance the membrane fouling resistance by incorporating the original membrane material with one (or more) nanofiller that possesses anti-fouling properties, such as silver, silica, zeolite, or a carbon nanotube, etc. [1]. These inorganic–organic hybrid membranes possess improved properties and performance compared to the original membranes, such as improved hydrophilicity and permeability, enhanced separation efficiencies or proton conductivity, and enhanced chemical stability or mechanical strength, etc. [1,2]. Such improvements often come directly from the properties of the nanoparticles, i.e., a hydrophilic material would bring more hydrophilicity to the membrane [3,4]. They can also come from a change in membrane morphology as the nanoparticles have an influence on the membrane formation process [5], or from chemical alterations such as the dynamic crosslinking interactions between the inorganic and organic phases [6,7].

Moreover, if the nanofillers also possess special properties, such as photocatalytic activity, the resulting composite membrane becomes even more effective in its operation. Additionally, among the common photocatalysts, titanium dioxide (TiO_2_) is one of the most widely used nanoparticles, as it exhibits a strong photocatalytic activity under ultraviolet (UV) irradiation, has a high availability as well as stability, yet a low environmental impact, and is relatively cheap. Pristine TiO_2_ is widely applied in the field of water treatment as it can release one of the strongest oxidants, the hydroxyl radical, which is very effective in degrading harmful and toxic pollutants [8]. Therefore, this property is particularly useful when used in combination with membrane processes. In fact, several configurations are possible for the coupling of photocatalysis with separation processes, including photocatalysis with membrane filtration, where the photocatalytic reactor and the filtration module are separated [9]. However, the photocatalytic membrane reactor configuration, where the photocatalyst is bound to the membrane, received growing interest due to its enhanced process intensification, while there are fewer concerns for the recovery or release of nanoparticles [10]; this is the reason why composite polymer-TiO_2_ membranes recently attracted much attention from researchers. Indeed, these composite membranes usually exhibit superior properties to the neat polymeric membranes, including a higher surface hydrophilicity, stronger permeate flux, improved fouling resistance, and antibacterial properties [11,12,13,14].

However, the drawback of including TiO_2_ photocatalysis in polymer materials is that it may lead to an unwanted degradation of the polymers, as many polymers are unstable under long-term exposure to UV irradiation as well as the oxidative environment caused by photocatalysis. Therefore, selecting a suitable polymer material for the composite membrane is a very important task. Polyvinylidenefluoride (PVDF) is one of the most common membrane materials due to its excellent mechanical, thermal and chemical properties [15]. More importantly, it is appropriate for photocatalytic composite membranes because PVDF shows one of the strongest resistances to UV irradiation among common polymer materials [16]. Thus, the composite PVDF-TiO_2_ membrane is a great choice among the various types of composite polymeric membrane currently being developed. The PVDF-TiO_2_ membranes can be used in many water processing applications, such as natural organic matter removal [17], degradation of pollutants [18], antibacterial applications [12] or membrane distillation [19].

The composite PVDF-TiO_2_ membrane can either be a mixed-matrix membrane, where TiO_2_ nanoparticles are entrapped inside the polymer matrix, or a surface-modified membrane, where TiO_2_ nanoparticles are only coated on its active layer. Of these two configurations, the latter poses more environmental impacts as weak-binding catalyst nanoparticles could be released along the stream, while the ability of the membrane to retain its photocatalytic activity over time is also uncertain [20]. Mixed-matrix membranes are simple to prepare and more reliable in their ability to retain photocatalysts, and thus the photocatalytic properties of the membrane over time. Additionally, in this case, the TiO_2_ nanoparticles also serves as an additive during the membrane formation process, which may help improve the intrinsic properties of the membrane, even before the photocatalytic aspect comes into play. To prepare mixed-matrix PVDF-TiO_2_ membranes, the non-solvent-induced phase separation wet-process (NIPS wet-process) is widely employed, in which the TiO_2_ nanoparticles are mixed along with the polymers and solvents, as well as any other additives, to form a dope solution appropriate for membrane casting and immersion precipitation.

Because PVDF is a semi-crystalline polymer, the formation process of PVDF-based membranes is quite complicated, leading to different final membrane properties depending on the preparation conditions. Especially for the composite PVDF-TiO_2_ membranes, the large variations in their filtration performance was previously demonstrated in the literature. Mericq et al. [21] pointed out that there were inconsistent results, in terms of permeability and hydrophilicity, when comparing the performance of polymer-TiO_2_ membranes in various studies. For example, sometimes authors saw great improvements in permeate flux with the addition of TiO_2_ to the membranes, while in other cases little or no improvement was observed. This could be attributed to the differences in membrane materials (usually PVDF, polysulfone, polyethersulfone or some co-polymers), the choice of solvents (usually dimethylacetamide, n-methyl-2-pyrrolidone or dimethylformamide) and pore-forming additives (usually polyethylene glycol, polyvinylpyrrolydone or LiCl) or the preparation method (NIPS, temperature-induced phase separation (TIPS) or sol–gel). However, even when the basic parameters, such as chemicals and methods, are the same, there are still elements of the membrane preparation procedure that can affect the end products, such as polymer concentration, the mass ratio of TiO_2_ to the polymer, ambient humidity, types of support or temperature. Additionally, there is a lack of understanding in the literature of the link between membrane preparation parameters and their properties, as well as functionalities. One notable parameter, which was addressed in our previous study, is the concentration of TiO_2_ in PVDF-TiO_2_ composite membranes prepared by the NIPS method. Investigation over a range of wt% TiO_2_/PVDF showed that an optimum value of around 20–25 wt% yielded the best membrane permeability and hydrophilicity, as beyond this value TiO_2_ nanoparticles tended to agglomerate inside the pores, causing a negative effect on pure water flux [21].

Nevertheless, one parameter that did not receive suitable attention during composite membrane preparation via NIPS is temperature, despite it being a predominant one. For example, in most cases the membranes were said to be prepared at room temperature, but that term itself may cover a range of temperature from 15 to 30 °C depending on the ambient conditions. More importantly, temperature was often controlled in discontinuity across each step of the NIPS procedure, whereas the casting device, the dope solution and the coagulation bath temperatures all played a role. Indeed, in the case that a temperature gradient exists between the polymer solution and the coagulation bath, TIPS and NIPS effects may interplay, complicating the membrane formation process and making the final membrane structure less predictable [22]. Therefore, it is important to pay attention to temperature variations during membrane preparation, from the temperature of the dope solution to the coagulation bath, as well as the casting device, to ensure minimal interferences, which may affect the final membrane structure and performance. Although some authors did study the effects of the coagulation bath temperature on neat PVDF membranes [5,23,24,25,26], the results are still inconsistent and little correlation was found between the membrane morphology and its performance. Generally speaking, when liquid–liquid demixing is favored over solid–liquid demixing (polymer crystallization), the membrane is often predicted to have a more porous structure. Additionally, when the precipitation rate is low, smaller void structures beneath the membrane skin layer can be observed [27,28]. Lee et al. [29] reported a trade-off between thermodynamic enhancement and kinetic hindrance during the phase separation of polysulfone membrane preparation when additives were added. Unfortunately, temperature has an influence on both the thermodynamic and kinetic aspects during membrane formation via phase separation, so it is not easy to determine which one is the dominant parameter in predicting the final membrane morphology.

Given the gap of understanding on the role of temperature during membrane preparation via NIPS, especially for PVDF and composite PVDF-TiO_2_ membranes, in this work, we aim to study how temperature influences the membrane formation process and the final structure and properties of these membranes. One unique point of this study is that the temperature was synchronized across each step of the preparation procedure, i.e., the temperature was consistent, with values of 10, 25, 50 or 70 °C for the polymer solution, casting device and coagulation bath. This was to limit any unwanted thermal-induced effects that may arise from a temperature discrepancy between the components of the membrane formation process. Then, the thermodynamic and kinetic aspects during membrane formation at these temperatures were examined by several experimental methods, and the final membrane structures and properties (permeability, porosity, thermal and mechanical properties, etc.) were characterized using a range of standard membrane characterization techniques. It should also be noted that, since, in this work, we focused only on the intrinsic properties of the PVDF-TiO_2_ membranes, their photocatalytic properties are detailed elsewhere. For example, studies on the distribution of TiO_2_ in the membranes, their photo-induced hydrophilicity and photocatalytic performance under UV conditions can be found in our previous works [21,30,31] and will surely be explored in future studies. Therefore, the results obtained from this work are expected to further contribute to the understanding of membrane formation via NIPS, especially the impacts of temperature. In addition, the temperature is a processing parameter that is relatively simple to adjust, and thus helps to provide a relatively easy way to tune and adapt the membrane properties depending on the requirements of the applications.

## 2. Materials and Methods

### 2.1. Materials

Polyvinylidenefluoride (PVDF) pellets with an average molecular weight (M_w_) of 275 kDa were used as the polymer material. The solvent was N-N-Dimethylacetamide (DMAc, assay > 99.5%) while the additive was Polyethylene glycol (PEG200, average M_w_ 200 Da). The nanoparticles were Aeroxide^®^ TiO_2_ P25 nanopowder (approx. 85% anatase and 15% rutile, size of c.a. 21 nm, assay > 99.5%). All chemicals were purchased from Sigma Aldrich (Darmstadt, Germany) and used as received.

### 2.2. Membrane Preparation

Composite PVDF-TiO_2_ membranes were prepared by the non-solvent-induced phase separation (NIPS) wet-process. The dope solutions were prepared by mixing specific amounts of PVDF (20 wt%), TiO_2_ (20 wt% TiO_2_/PVDF, i.e., the TiO_2_ concentration was expressed as the ratio of TiO_2_ mass to PVDF mass), PEG200 (5 wt%) in DMAc. The mixture was sonicated for 20 min and then stirred for 24 h at 50 °C in a magnetic stirrer to ensure a homogeneous solution was obtained. Afterwards, the solutions were adjusted to desired temperatures (10, 25, 50 and 70 °C) by means of a hot plate or a cryostat. The solution was then cast on a Teflon sheet which was taped on a glass plate, using an automated casting knife (Erichsen, Hemer, Germany) set at 250 μm thickness and 50 mm s^−1^ velocity. The temperature of the glass plate was controlled by a heating plate or, in case of low temperature, by placing it inside a refrigerator prior to casting, while the temperature at the surface of the Teflon sheet was monitored by an infrared thermometer (Testo 845, Testo, West Chester, PA, USA) so that it was equal to the temperature of the dope solution at the moment of casting. After casting, the plate was immediately immersed in a deionized water bath prepared at the same desired temperature (the exposure time of the cast film to ambient air before immersion was less than 10 s). In all experiments, the temperature of the dope solution, the casting plate and the coagulation bath were thus set at the same value (10, 25, 50 or 70 °C). The membranes were left in the coagulation bath for 12 h, then detached from the Teflon support, rinsed thoroughly with deionized water and stored in milliQ^®^ water (18 MΩ.cm resistivity) at room temperature under dark condition. For neat PVDF membranes, the procedure was exactly the same as for PVDF-TiO_2_ membranes, except that no TiO_2_ was added to the dope solutions.

Viscosity of the casting solutions was measured by a temperature-controlled rotational rheometer (Physica MCR 301, Anton Paar, Graz, Austria) with a 50-mm-diameter, 1° cone. The final values were taken by averaging the viscosities from shear rate of 100 to 101 s^−1^.

### 2.3. Study of Phase Separation Thermodynamics via Ternary Phase Diagrams

The ternary phase diagrams were established for two systems (PVDF/DMAc,PEG/H_2_O and PVDF/DMAc, PEG, TiO_2_/H_2_O) at four temperatures (10, 25, 50 and 70 °C), using a procedure based on the cloud point titration method. First, a series of homogeneous, identical polymer solutions (10 or 20 wt% PVDF) were prepared using the procedure previously described. Then, specific amounts of water, ranging from 1 to 15 wt%, were added to each solution, making the local precipitates appear. The solutions were then heated to 80 °C under strong agitation until the precipitates disappeared and the solutions became homogeneous again. Afterwards, they were cooled down to the desired temperatures (10, 25, 50 and 70 °C) and maintained overnight. The cloud point was identified as the composition where a minimum amount of added water could induce turbidity/gelation to the polymer solution at a given temperature. The binodal lines at each temperature were created by connecting the corresponding cloud points of the systems at that temperature.

### 2.4. Study of Phase Separation Kinetics via Light Absorbance

The phase separation kinetics study via light absorbance is based on the principle that when phase separation progresses, the casting solution gradually loses its transparency, in turn increasing the light absorbance measured by a spectrophotometer. Consequently, this method could not be applied to a casting solution containing TiO_2_, because TiO_2_ nanoparticles would make it opaque, thus not allowing light transmission. Therefore, a thin layer of PVDF casting solution (without TiO_2_) was cast on a small glass slide, which was immediately placed vertically in a plastic cuvette containing deionized water at a given temperature (the dope solution and the glass slide were pre-heated to that temperature as well). The light absorbance was then monitored over time by a spectrophotometer (UV-2400PC, Shimadzu, Kyoto, Japan) at 300 nm, which was the wavelength that provided the strongest absorbance for the type of PVDF solution in this study. The derivatives of the absorbance curves were performed to represent the increased rate of absorbance.

### 2.5. Microscopic Observation of Water Entrance during NIPS

To imitate the membrane formation process by non-solvent addition, a drop of the polymer solution was placed between a glass slide and a cover slip under an optical microscope (BX41, Olympus, Tokyo, Japan). The drop was thinned and left to spread so that it reached the edge of the cover slip, where a drop of water was introduced. Upon contact with water, phase separation occurred as water started to penetrate the polymer solution spread between the slides. This process was observed by a 50× objective lens and recorded using a digital camera at 29 fps. The experiments were performed at two temperatures: 25 and 70 °C, with temperature of the polymer solution and water controlled by a heating plate, while the temperature of the microscopic slide was maintained by a heating and freezing stage system (LTS350, Linkam, Tadworth, UK). The void growth rates were then compared by measuring the evolution of water penetrating the polymer solution with the aid of binary image conversion and pixel counting tools from the software ImageJ (v1.46r, NIH, Bethesda, MD, USA) and MATLAB (R2017a, MathWorks, Natick, MA, USA).

Although the visualization technique for phase separation by optical microscope has a few shortcomings (for example, the water is introduced to the polymer solution horizontally, instead of vertically, as in the real case of immersion precipitation, while the thickness in membrane casting is much lower than the length of a spread polymer solution drop), it is still useful for providing additional and relative information on the void formation process of a membrane during phase separation [32].

### 2.6. Membrane Structural Characterization

The surface and cross-sectional morphology of membranes were examined by scanning electron microscopy SEM (S4800, Hitachi, Tokyo, Japan). Cross-sectional samples were prepared by cryo-breaking the membranes in liquid nitrogen. All samples were dried at 35 °C and coated with a sputtered platinum layer prior to SEM analyses.

Membrane porosity was determined by a classic gravimetric method [33]. Membrane samples were immersed in deionized water for 24 h and weighed after mopping out all the water on the surface. Then, the samples were dried in an oven at 35 °C to constant weight and weighed again. The total porosity of the membrane was calculated as:

ε = (W_w_ − W_d_) × 100/(ρ_w_Aδ) (1)

where ε is the porosity of the membrane (%); W_w_ and W_d_ are the wet weight and dry weight (g), respectively; ρ_w_ is the density of pure water (g cm^−3^); A is the area of the membrane sample (cm^2^) and δ is the wet thickness of the membrane (cm), which is measured by a digital micrometer (Mitutoyo, Kanagawa, Japan).

### 2.7. Thermal and Crystalline Properties

The thermal properties of membranes were characterized by differential scanning calorimetry (DSC-Q20, TA Instruments, New Castle, DE, USA). Samples were heated in sealed aluminum pans from 0 to 230 °C at a heating rate of 10 °C/min under a 50 mL/min flow of nitrogen gas. The total crystallinity (X_c_) of PVDF was calculated by the equation [34]:

X_c_ = (ΔH_f_)/(w × ΔH_f_^0^ ) × 100% (2)

where ΔH_f_ and ΔH_f_^0^ are the heat of fusion of the samples and of an ideal PVDF crystal, respectively (ΔH_f_^0^ = 105 J/g [35]), and w is the weight fraction of PVDF in the samples (which is 1 for neat PVDF membrane and 0.83 for PVDF-TiO_2_ membranes). The heat of fusion of the samples (ΔH_f_) is determined by integrating the areas under the melting peaks.

Determination of the crystalline forms of PVDF was performed using Fourier-transform infrared (FTIR) spectroscopy (Nexus, Thermo Scientific, Waltham, MA, USA) in ATR-FTIR mode. Samples were scanned in the wave number range of 4000–650 cm^−1^ with a resolution of 2 cm^−1^.

### 2.8. Membrane Permeability and Mechanical Properties

Membrane permeability was measured with deionized water at room temperature using a dead-end ultrafiltration system with a 50 mL membrane cell (Amicon, Merck Millipore, Burlington, MA, USA). Pressure was provided by compressed air in the range of 0–2 bar. Permeate flux was determined by monitoring the permeate mass via an electronic balance connected to a computer. Prior to each measurement, the membrane underwent compaction period comprising of 15 min water filtration at 0.25, 0.5, 0.75 and 1 bar successively, and then 30 min at 1.25 bar. The feed temperature was monitored throughout the experiments and all permeabilities were expressed at 20 °C.

The mechanical property of the membrane was characterized by a texturometer (TAXT2i, Swantech, Gennevilliers, France) with a pulling rate of 0.5 mm s^−1^. The tensile strength was calculated based on the force at the break of the samples.

## 3. Results and Discussion

In this study, our first aim is to examine the differences in the thermodynamic and kinetic aspects of the membrane formation process at different temperatures, along with an observation of the non-solvent (water) entrance once the polymer is in contact with water, all of which determine the properties of the formed membranes. Once the actual membranes were created and characterized, we attempted to explain their properties based on the information obtained from the first part, while also establishing a link between their performance and properties.

### 3.1. Phase Separation Thermodynamics

**Figure 1:**
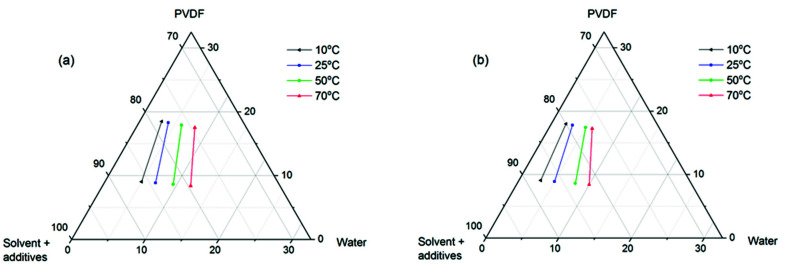
Phase diagram of (**a**) PVDF/DMAc,PEG/H_2_O and (**b**) PVDF/DMAc,PEG, TiO_2_/H_2_O systems.

The ternary phase diagram is typically used to describe the thermodynamics of a polymer/solvent/non-solvent system. In this study, the experimental binodal curves were constructed by connecting the two cloud points determined via the method presented in Section 2.3. It is well-reported in the literature that both liquid–liquid demixing and solid–liquid demixing/crystallization can be responsible for the formation of phase separation membranes [28]. Since the cloud point method cannot quantify how fast the composition of the system changes, the cloud points here might result from both liquid–liquid and solid–liquid demixing of the polymer solution, especially for a semi-crystalline polymer such as PVDF. In Figure 1, a phase diagram at four temperatures of PVDF-based systems without (Figure 1a) and with TiO_2_ (Figure 1b) can be seen.

It is well-established in the literature that the addition of additives to a polymer solution generally moves the binodal curves towards the polymer/solvent axis, as the additives act as anti-solvents, reducing the solvent strength and system tolerance for non-solvents [36,37]. This rule is also reflected in our cases. At each temperature, the corresponding binodal curves shift leftwards when TiO_2_ is present in the system, as this hydrophilic additive lowers the thermodynamic stability of the systems. More importantly, regardless of the presence of TiO_2_ in the casting solutions, a gradual shift of the binodal curves towards the polymer/solvent axis can be seen when the temperature decreases from 70 to 10 °C. Therefore, at lower temperatures, the casting solutions are closer to their precipitation state, thus requiring less water to be thermodynamically disturbed. However, it should be noted that the ease of disturbing a polymer system does not necessarily correspond to the rates of phase separation or polymer precipitation. As generally reported in the literature, a high polymer precipitation rate often leads to an asymmetric membrane morphology with macrovoids and/or finger-like cavities beneath the skin layer [27,28] and, in some cases, researchers tended to associate phase diagrams that had a small miscibility gap (the distance between binodal curve and polymer/solvent axis) with a fast phase separation [26,38]. In our opinion, such an assumption, while being valid in certain cases, is an oversimplification of the whole membrane formation process and the kinetic aspect of phase separation must also be considered.

### 3.2. Phase Separation Kinetics

**Figure 2:**
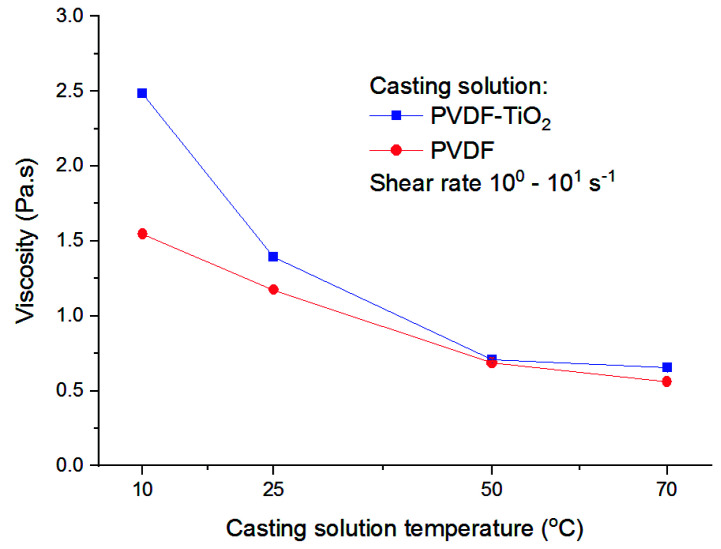
Viscosity of DMAc−based casting solutions at different temperatures.

Viscosity is a basic parameter in the membrane formation process, as it plays an important role in the kinetic aspects of phase separation. Figure 2 presents the viscosity of both types of casting solution, with or without TiO_2_, measured at different temperatures.

It is apparent that at lower temperatures of 25 °C, and especially 10 °C, the viscosity of PVDF-TiO_2_ solutions was higher than that of PVDF solutions. This result is easy to understand, given that the former is essentially a suspension containing well-dispersed TiO_2_ nanoparticles in a polymer solution, while the latter is only the dissolved polymer solution itself. However, at higher temperatures (50 and 70 °C), the viscosities of solutions with and without TiO_2_ are almost the same, suggesting that the influence of temperature is much stronger than the influence of the presence of nanoparticles in the solutions. Nevertheless, a gradual decrease in viscosity with temperature was expectedly observed, which was due to the increasing molecular movements in the solutions at higher temperature. For PVDF-TiO_2_ solutions, the viscosity showed an almost four-fold decrease from 2.5 Pa.s at 10 °C to 0.65 Pa.s at 70 °C.

**Figure 3:**
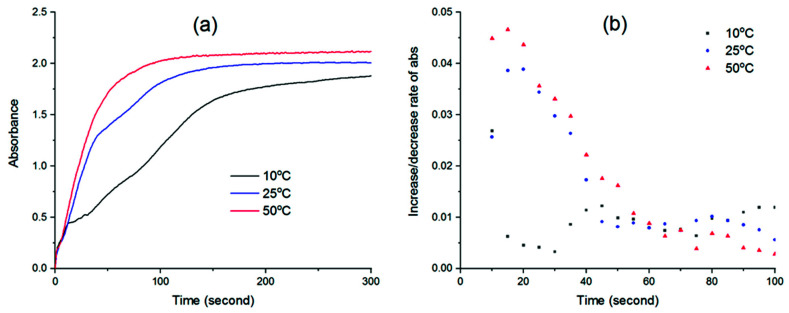
(**a**) UV absorbance over time of PVDF films immersed in water and (**b**) its increase rate.

The kinetics of phase separation during NIPS can be represented by the rate of solvent–nonsolvent exchange after the cast film is immersed in the coagulation bath. Thus, the degree of translucence of the cast film over time is an indicator of the speed at which phase separation progresses [39,40]. The evolution of UV absorbance with time and its increase rate (d Abs/dt) after the immersion of the PVDF thin films in water at different temperatures can be seen in Figure 3.

From Figure 3a, an immediate increase in absorbance could be seen in the first few seconds after the cast films were immersed in water. This was due to light absorption effect of the original films itself, which appeared the same at 10, 25 or 50 °C. Therefore, regardless of temperature, little differences can be observed between the three curves at the beginning. Only after the first 10 s, the distinctive features of each absorbance curve can be seen. Since the mass transfer rate depends on temperature, the exchange of water-solvent at different temperatures will occur at different rates. As phase separation occurs, the newly cast films will gradually solidify and lose their transparency over time, thus increasing the UV absorbance. Once the polymer completely solidifies, the absorbance stops increasing and reaches a stable state. Here, it can be seen that the absorbance curve obtained at the highest temperature (50 °C) reaches a plateau the soonest at 100 s after immersion, while the curves at 25 and 10 °C only become stable after about 150 s and 300 s, respectively. This suggests that the water–solvent exchange process at a higher temperature is faster. Indeed, as can be seen in Figure 3b, the increase rate of absorbance at 50 °C was always highest for the first 50 s, followed by the rate at 25 °C, while that at 10 °C was much slower. After that, the absorbance at 50 °C approached the stable phase, explaining why its increase rate gradually approached zero. Since this rate is proportional to phase separation kinetics, the demixing rate of the PVDF solution and water increases with membrane preparation temperature, and it is expected that the PVDF-TiO_2_ solution follows the same rule. In fact, there was a correlation between solution viscosity and film absorbance as, when the solution was most viscous at low temperature, the demixing rate was slowest and vice versa. It is a common understanding in phase separation membrane theory that instantaneous demixing favors a more porous structure with finger-like macrovoids, while delayed demixing often yields a sponge-like structure. In our case, it is clear that, with the increase in temperature, phase separation kinetics are enhanced due to the lower solution viscosity and higher solvent–nonsolvent exchange rate, yet the solution is thermodynamically more stable, as analyzed in the previous section. These competitive effects make it difficult to predict the final membrane morphology simply based on the rules generally reported in the literature. Thus, more insights on the membrane formation process from this particular system are needed to explain the final membrane structure.

### 3.3. Microscopic Observation of Membrane Formation

**Figure 4:**
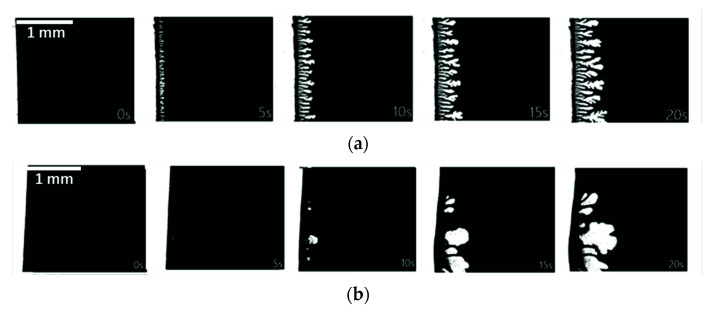
Binarized image series of water entrance to PVDF-TiO_2_ solution at (**a**) 25 °C and (**b**) 70 °C.

The direct observation of phase separation via an optical microscope is a useful technique to monitor the real-time progress of water entrance into a polymer solution. This method was verified by several authors in the study of macrovoids formation in membranes [32,41]. In our case, the white TiO_2_ nanoparticles in the PVDF-TiO_2_ polymer solution conveniently serve as a tracer that makes the penetration of water into this solution much easier to visualize, compared to the case of water penetrating into the transparent PVDF solution, where the contrast is not strong enough for an observation. For this reason, the experiment was only performed on the PVDF-TiO_2_ solutions. Figure 4 compares the entrance state of the non-solvent to the polymer solution (only the first 20 s shown) when water is in contact with the edge of a PVDF-TiO_2_ thin film at two temperatures, 25 and 70 °C.

**Figure 5:**
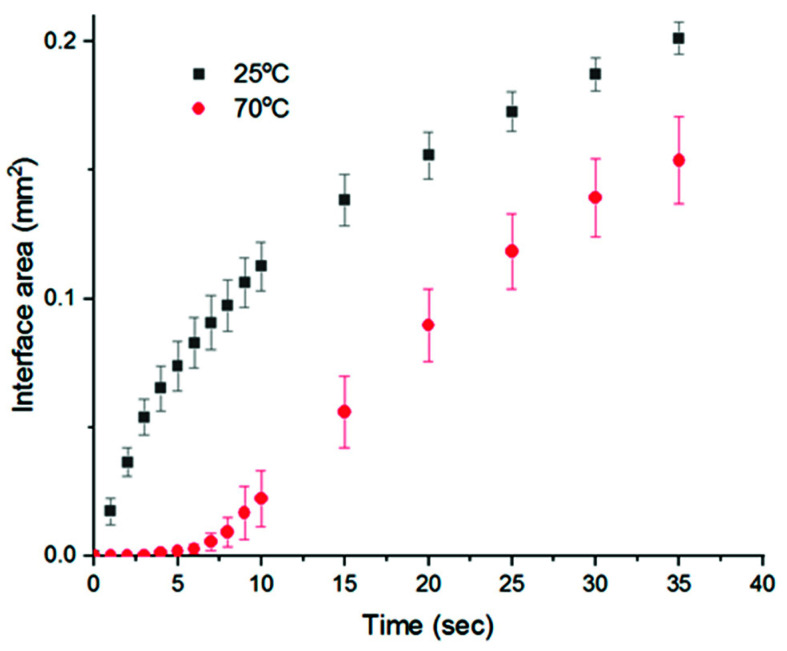
Comparison of macrovoid growth rate at 25 and 70 °C.

After the binarization of the microscopic image sequences, the white areas represent the areas caused by water penetration into the polymer solution, which correspond to the polymer-lean phase, and thus the membrane macrovoids; while the black areas correspond to the polymer-rich phase, and thus the membrane matrix. The void growth rate was reflected by the increase in the interface area, which represents the length of the boundary between the penetration front and the polymer solution. It can be observed that, at 25 °C, upon the contact with the polymer solution, water started to penetrate into the solution almost immediately, with a uniform growth in a perpendicular direction to the initial water–polymer solution interface (Figure 4a), while at 70 °C the entrance of water appeared to be more difficult and could only start after a delay between 5 to 10 s (Figure 4b). The lower viscosity of the polymer solution at 70 °C, compared to 25 °C, might explain why, once water was able to disturb the interface, the penetration fronts expanded very rapidly in arbitrary directions inside the polymer solution, as the effect of kinetic hindrance in this case was lower [29], allowing an easier exchange between water and solvent. Quantitatively, Figure 5 confirms that, despite the delayed water entrance at 70 °C, after the first 10 s, the interface area growth rate was comparable to that at 25 °C. From these observations, it appears that the development of water in the polymer solution °C occurred differently between the two stages: (i) the entrance at the water–polymer solution interface during the first few seconds, and (ii) once water already penetrated the polymer solution through the interface. In the first stage, the interface area growth rate at 70 °C was much slower compared to that at 25 °C. This can be explained by the denser top layer of the 70 °C solution due to the faster evaporation of the solvent. However, in the second stage, once the water was able to pass this dense layer, expanding into the bulk of the solution via the already established entrance sites, the restriction at the top layer became less important, while the lower viscosity of the solution at 70 °C may become more significant. Thus, the interface area growth rates between the two solutions (25 and 70 °C) become comparable. While this information on void growth rate may be useful in predicting the final membrane morphology, one should not simply, from Figure 5, correlate the smaller interface area at 70 °C with smaller macrovoid structures in the final membrane morphology, especially when considering that the microscopic observations of void growth were performed at a distance almost 10 times higher than that of the membrane casting thickness, i.e., the longer the distance, the greater the differences in the microscopic observations and real membrane forming process.

In order to link these results with the actual membrane formation process, it is more interesting to focus on the entrance of water during the earlier stage, when the length of the water penetration front is only marginally higher than the thickness of a cast film in real membrane preparation situations. Indeed, a stark feature distinguishing the two processes at 25 and 70 °C is the number of initial sites for water entrance, which corresponds to the initiation of macrovoid growth. Here, it should be first mentioned that the development of water from these entrance sites is related more to the formation of finger-like structures in membrane morphology, rather than an indication of the phase separation rate. This process should not be mistaken with the general water–solvent exchange during phase separation, as water can in fact be exchanged with solvents in the polymer solution without creating distinctive evidence of those finger-like patterns under the magnification of the optical microscope [32]. Comparing the images at the point of 10 s in Figure 4a,b, it is clear that at 25 °C the initial sites for water entrance can be seen abundantly across the water–polymer solution interface, while at 70 °C only a few sites can be observed. Over time, for the case at 70 °C, water only continued to penetrate the polymer solution via these sites, without being able to further disturb the interface and create new sites for water entrance. Therefore, it can be predicted that the earlier and easier entrance of water at 25 °C would allow the formation of more finger-like macrovoids in the membrane structure. However, at 70 °C, prior to the introduction of water, it seemed that the polymer near the interface was partly solidified, making it harder to disturb and leading to the lack of initial sites for water entrance. Thus, the occurrence of finger-like macrovoids in membranes prepared at high temperatures may be more difficult. As a result, this hypothesis is further examined by exploring the cross-sectional morphology of the membranes via SEM, as described in the following section.

### 3.4. Membrane Structure and Properties

**Figure 6:**
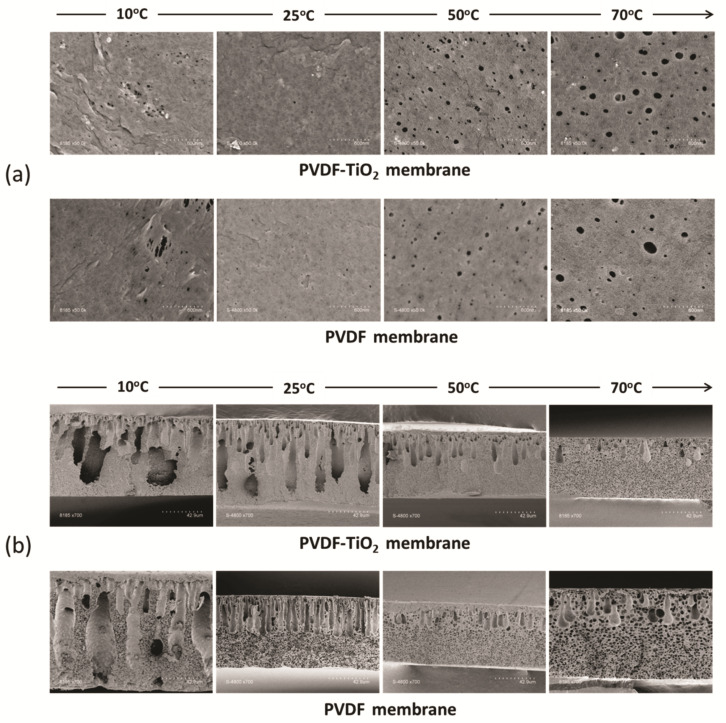
SEM images of PVDF-TiO_2_ and PVDF membranes, (**a**) top surface and (**b**) cross section.

The structure of asymmetric membranes prepared by NIPS is well-established in the literature. Typically, the membrane contains a very thin, “dense” top layer serving as the separation layer, followed by a more porous structure, which may contain some finger-like macrovoids, on top of a supporting microporous layer that provides the membrane strength. In our case, both the structures of PVDF-TiO_2_ and PVDF membranes followed this rule, as shown in Figure 6.

The membrane active surfaces made at various temperatures can be seen in Figure 6a. The effect of temperature can clearly be seen in both cases, for neat PVDF, as well as for PVDF-TiO_2_ membranes. At lower temperatures (10 and 25 °C), membrane surfaces appeared denser, whereas at high temperatures (50 and 70 °C) there was a significant increase in both the number of pores and surface pore size, with the highest pore diameter up to 60 nm for PVDF-TiO_2_ membranes prepared at 70 °C. The quick influx of non-solvent after immersion of the cast film is probably responsible for this increase, since at higher temperatures the exchange rate is higher, as demonstrated above. It is also apparent that the surface pore density of the membranes with TiO_2_ was higher than the ones without TiO_2_ at the same temperature. This can be explained by the presence of TiO_2_ nanoparticle sites on the interface, which attract more water influx due to their hydrophilic properties.

In Figure 6b, the cross-sectional morphology of membranes can be seen. It appears that, regardless of the addition of TiO_2_, a mutual trend of morphology transition can always be seen when the membrane preparation temperature increases. Indeed, a gradual decrease in both the number and size of finger-like structures can be observed. At lower temperatures (10 and 25 °C), these finger-like cavities often occupy a large area throughout the membrane thickness, sometimes almost extend to the bottom of the membranes. Then, they gradually shrink in size or decrease in number with temperature, to the point that a bicontinuous structure becomes the dominant morphology (membrane at 70 °C). The membrane morphology at higher temperatures correlates with the hypothesis on macrovoids formation proposed in Section 3.3. While large cavities in membranes are often associated with a better passage through which water can pass, they may also weaken the membrane mechanical strength as these structures are easier to collapse under high pressure. Thus, given these surface and cross-section morphologies, the membranes prepared at lower temperatures were expected to have a better separation rate and higher permeate flux at the same time, while being weaker in terms of mechanical strength.

One of the most complicated aspects of phase separation membranes is the study of macrovoids formation and the transition from finger-like macrovoids to microporous spongy structures (or vice versa). While authors were able to set general rules on how membrane preparation conditions affect the formation of macrovoids, more often than not they are only applicable to the specific systems investigated in those studies, while exceptional cases are also not uncommon. Various mechanisms of macrovoids formation have been proposed to date, as can be seen in Table 1. Nevertheless, macrovoids formation most likely cannot be attributed to one single mechanism, as it involves several different processes occurring at the same time.

In addition to the more popular mechanisms summarized in Table 1, several new observations on macrovoids formation were reported recently, including the growth of macrovoids parallel to the membrane skin [52]; the formation of a polymer lean layer at the phase-separating boundary, which led to macrovoids formation [53]; the formation of macrovoids due to non-solvent intrusion through the bottom surface of the cast film [54] or the effect of polymer chain entanglement on the initiation of macrovoids [55]. Generally, without a special focus on the macrovoids formation mechanism, most authors used the demixing theory to explain NIPS membrane morphology, in which a fast demixing rate was generally associated with the dominance of finger-like macrovoids and delayed demixing was associated with sponge-like structures. However, contrary evidence was also reported where instantaneous demixing was demonstrated to be insufficient to facilitate macrovoids formation [48,50,56]. Additionally, in case the membrane preparation parameter has dual but competitive effects on both thermodynamic and kinetic aspects of phase separation, it is not easy to draw a conclusion. In our case, the problem was further complicated due to the influence of both additives and temperature, as well as the semi-crystalline nature of PVDF. Here, the more recently proposed mechanism of viscous fingering may be able to explain our results. Originating from a fluid replacement study in porous media, viscous fingering is a phenomenon observed when a less viscous fluid replaces a more viscous fluid, eventually leading to the propagation of the less viscous fluid and the formation of complex finger-like patterns [51]. Therefore, the nature of viscous fingering is very similar to the exchange of water (the less viscous fluid) and solvent (the more viscous fluid) in the phase separation process. Ren et al. [50] reported that when the viscous fingering rate was faster than the rate of the phase separation front, finger-like structures were more favored and vice versa. In our case, the propagation of water in the polymer solutions, which corresponds to the viscous fingering rate, was identified as easier at lower temperatures during the initial period (Figure 4). Additionally, the rate of phase separation at higher temperatures was higher, as demonstrated in the kinetics study (Figure 3). Therefore, it could be argued that at higher temperatures (50 and 70 °C) the viscous fingering rate was not sufficient to outlast the phase separation rate, leading to a decrease in the size of the finger-like structures, and the bicontinuous structures became more dominant. In addition, as discussed in Section 3.3, at 70 °C there were fewer initial sites for water entrance when the nonsolvent was in contact with the cast film, possibly due to the partly solidified layer near the interface, which was also correlated with the faster phase separation kinetics at high temperature. This occurrence of a skin layer that retarded water transfer through the cast film was also reported for membranes prepared by vapor-induced phase separation (VIPS) [57]. In our case, the solvent outflow rate during the few seconds when the cast film was exposed to air prior to immersion was probably higher at 70 °C, which might also contribute to the fast polymer shrinking near the interface and support the formation of this layer. Hence, the decrease in the number of fingers at higher temperatures is logical.

**Figure 7:**
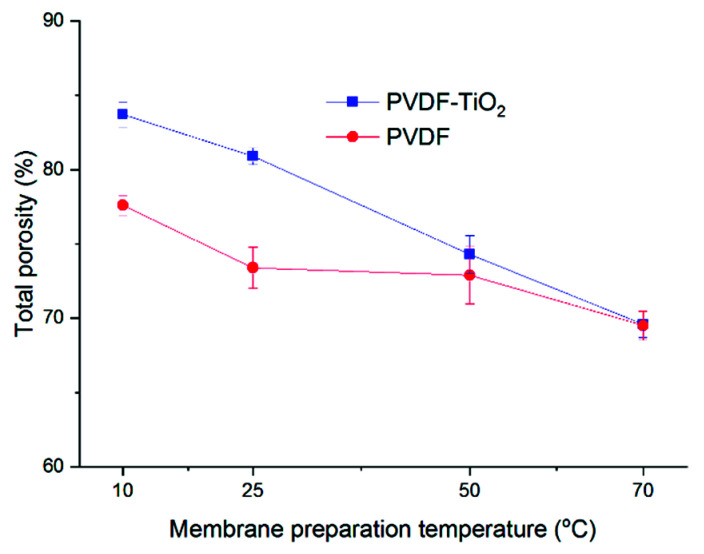
Membrane porosity.

Porosity measurements of the membranes further confirm the observations from cross-sectional SEM (Figure 7). The porosity of PVDF and PVDF-TiO_2_ membranes both decreased upon the increase in temperature, from 83% at 10 °C to 70% at 70 °C for PVDF-TiO_2_ membranes, and from 78% at 10 °C to 70% at 70 °C for neat PVDF membranes. It could also be observed that, at lower temperatures (10 and 25 °C), the porosity of composite membranes was higher than that of the neat membranes, though at higher temperatures (50 and 70 °C) this difference seemed to be less noticeable.

### 3.5. Thermal and Crystalline Properties

**Figure 8:**
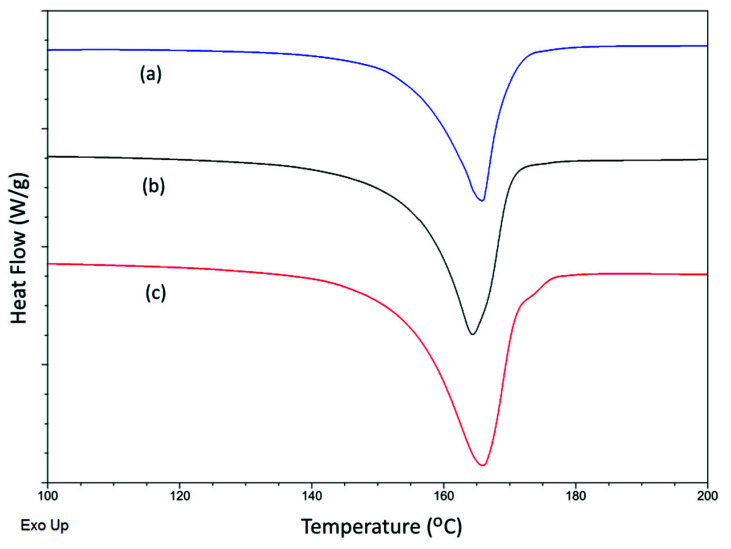
DSC thermograms of PVDF-TiO_2_ membranes prepared at (**a**) 70 °C and (**b**) 10 °C, and of (**c**) PVDF membrane prepared at 10 °C.

The DSC thermograms of selected membranes can be seen in Figure 8. All DSC curves show an endothermal peak around 166 ± 1.5 °C, which corresponds to the melting temperatures (T_m_) of PVDF. By normalizing the heat of fusion ΔH_f_ with ΔH_f_^0^, the total crystallinity of membranes (X_c_) was determined and summarized, along with T_m_, in Table 2. Although the variations are very small, it can be seen from Table 2 that T_m_ increased slightly with the membrane preparation temperature, and that PVDF-TiO_2_ membranes had lower T_m_ compared to PVDF membranes prepared at the same temperature. The opposite trend was observed for the X_c_ of both PVDF and PVDF-TiO_2_ membranes, as increasing the membrane preparation temperature tended to slightly reduced X_c_, although the difference was difficult to see between the two membranes prepared at similar temperatures (i.e., the decrease in X_c_ with the membrane preparation temperature can be seen more clearly if the membranes prepared at 10 and 70 °C, for example, are compared). The decrease in X_c_ with temperature can be explained based on the study of phase separation kinetics. When the temperature increases, the solutions become less viscous and phase separation kinetics is enhanced. Therefore, liquid–liquid demixing becomes more favored compared to crystallization, which leads to an eventual decrease in crystallinity. In addition, the presence of TiO_2_ (at 20 wt% to PVDF) in the membranes seemed not to affect X_c_, as the differences of X_c_ between the PVDF and PVDF-TiO_2_ membranes prepared at the same temperatures were also small and insignificant. This suggests that the influence of temperature on the membrane formation process, with respect to crystallinity, is much stronger than the influence of the additive.

**Figure 9:**
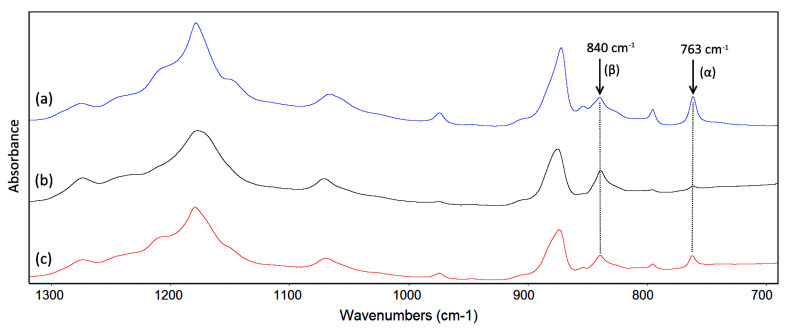
FTIR spectra of (**a**) PVDF membrane prepared at 25 °C, and PVDF−TiO_2_ membranes prepared at (**b**) 25 °C and (**c**) 50 °C.

FTIR spectroscopy is widely used to study the crystalline phases of PVDF. Selected FTIR spectra representative of the membranes in this study are shown in Figure 9. As extensively reported in the literature, the three main polymorphs of PVDF (α, β and γ) can be identified within the range of 1500–600 cm^−1^ in the FTIR spectra [58,59]. For these polymorphs, the exclusive peaks for the α phase are around 763, 795 and 975 cm^−1^; the exclusive peak for the β phase is around 1275 cm^−1^ and the exclusive peaks for the γ phase are around 811 and 1234 cm^−1^. Several authors often attributed the peak around 840 cm^−1^ to the β phase during the quantification of this individual phase, while in fact it can be characteristic of both electroactive phases β and γ. In our case, since the presence of the 1275 cm^−1^ peak and the absence of the 811 and 1234 cm^−1^ peaks are easily recognized in the spectra of all membranes, they are good evidence that the peak at 840 cm^−1^ can be assigned to only the β phase [59,60,61]. Hence, it can be considered that only two main polymorphs (α and β) were present in our membranes, and the quantification of the mass fraction of the α phase, based on IR absorbance, can be applied using the equation [62]:

(3) $$F_{\alpha }=\frac{A_{\alpha }}{(K_{\alpha }/K_{\beta })A_{\beta }+A_{\alpha }}\text{×}100\%$$

where F_α_ is the mass fraction of the α crystalline form in the PVDF crystals contained in the membrane; K_α_ (6.1 × 10^4^ cm^2^/mol) and K_β_ (7.7 × 10^4^ cm^2^/mol) are absorption coefficients of the α and β phases, respectively, and A_α_ and A_β_ are the IR absorbance of the characteristic peaks for α and β phases which are at 763 and 840 cm^−1^, respectively.

The fractions of the α crystallite in PVDF and PVDF-TiO_2_ membranes are listed in Table 2. It can be seen clearly that, for both types of membrane, the fraction of the α phase increased with the preparation temperature. For PVDF-TiO_2_ membranes, α crystallite was the dominant type when membranes were prepared at 50 and 70 °C, while β crystallite became more dominant at lower temperatures (10 and 25 °C). For PVDF membranes, β crystallite was dominant only when the membrane was prepared at the low temperature of 10 °C. The dominance of α and β phases at high and low temperatures, respectively, may be explained by the thermodynamic stability of each form. Since the β phase of PVDF is thermodynamically meta-stable, while the α phase is thermodynamically more stable, at higher temperatures, the low degree of entanglement and high mobility of PVDF chains would facilitate the formation of the α phase [26,59,63]. In addition, the gradual decrease in T_m_ with the membrane preparation temperature, as well as the lower T_m_ of PVDF-TiO_2_ membranes compared to PVDF membranes, may also be explained by the mass fraction of the β phase, as it was reported that the increase in the β phase could induce a decline in the melting temperature of PVDF [63]. Indeed, as in our case, the fraction of the β phase was higher when membranes were prepared with TiO_2_, as well as at lower temperatures.

### 3.6. Membrane Permeability and Mechanical Properties

**Figure 10:**
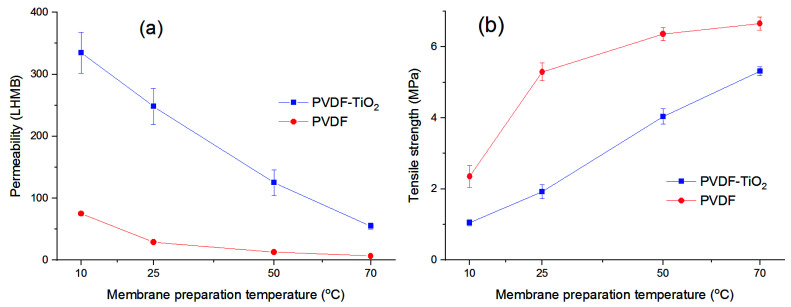
Membrane (**a**) permeability at 20 °C and (**b**) tensile strength.

The performance of PVDF-TiO_2_ membranes, in comparison with neat PVDF membranes, was characterized by permeability and mechanical strength measurements, and can be seen in Figure 10. As expected, the permeability of PVDF-TiO_2_ membranes was significantly higher than that of the neat PVDF membranes, especially at a lower temperature (Figure 10a), given that PVDF is a hydrophobic polymer. The increase in permeability was largely attributed to the hydrophilicity that TiO_2_ nanoparticles bring to the composite membranes [11,64]. More importantly, increasing the membrane preparation temperature induced a negative effect on its permeability, as a decrease in permeability from 330 LHMB at 10 °C to just 70 LHMB at 70 °C was observed for the PVDF-TiO_2_ membranes. The same trend applied for neat PVDF membranes, as permeability decreased from 75 to 10 LHMB for the same temperature rise. It is also worth noting that the permeability of the neat PVDF membrane prepared at 10 °C was slightly higher than that of the composite PVDF-TiO_2_ membrane at 70 °C, showing that the beneficial effect of TiO_2_ on membrane permeability could be mostly suppressed with temperature control during preparation instead of dope composition control. Nevertheless, these results are in agreement with the SEM image observations, as at a low temperature the formation of large finger-like macrovoids facilitates the quick passage of water through the membrane thickness, thus increasing the membrane permeate flux. It is also noted that porosity and surface pore size seem to play a smaller role in permeability in this case. Despite the bigger pore size and not much lower porosity, the membranes prepared at higher temperatures still exhibited a lower permeability compared to the membranes prepared at lower temperatures.

However, as a trade-off for permeability, the membranes prepared at lower temperatures showed a lower mechanical resistance, as can be seen in the tensile strength tests (Figure 10b). PVDF-TiO_2_ membranes prepared at 10 °C showed a tensile strength of 1.04 MPa, while the corresponding figure at 70 °C was more than five-fold, at 5.31 MPa. Similar to permeability but in an opposite way, the tensile strength of both neat and composite membranes gradually increased when the membranes were prepared at higher temperatures. This is not surprising given the cross-sectional structure of these membranes, as large finger-like cavities are an indication of a mechanically weak membrane, often deemed an undesirable property. As the temperature increased, these finger-like macrovoids decreased and the membranes became more compact, thus improving their tensile strength. It is also worth noting that, the addition of TiO_2_ at 20 wt% to the membrane matrix actually weakened its mechanical property, as the tensile strength of the neat PVDF membranes was higher than that of PVDF-TiO_2_ membranes prepared at the same temperature. This can be largely explained by membrane porosity (Figure 7). Because the composite membranes with TiO_2_ were always more porous than their neat PVDF counterparts, it is easier to break their structure by mechanical force, hence the reduced mechanical strength. In this case, the weakening influence of porosity may overcome any reinforcing effect on mechanical strength, caused by the nanoparticles, though this overlap might not necessarily happen at a different concentration of TiO_2_. In addition, another factor that may play a role in determining the membrane mechanical strength is the crystalline form of PVDF, as it was reported that the dominance of α crystallites contributed to the improvement of the mechanical properties of membrane [25]. As such, the increase in F_α_ with temperature, as well as the higher F_α_ in neat PVDF membranes may help explain the higher mechanical strength of PVDF membranes, as well as membranes prepared at higher temperatures.

**Figure 11:**
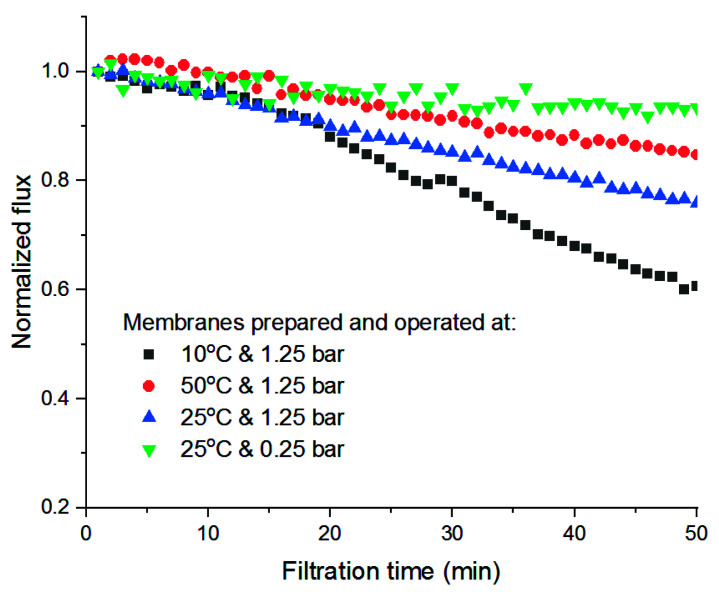
Flux decrease in PVDF-TiO_2_ membranes due to compression.

Finally, a comparison of the permeate flux decrease due to the compression at high pressure between PVDF-TiO_2_ membranes prepared at different temperatures is presented in Figure 11. It can be seen that the lower the preparation temperature, the more compressed the membranes became after being operated at a transmembrane pressure of 1.25 bar, reflected by the fact that their permeate flux declined at a stronger rate. For example, after 50 min, the flux of the membrane prepared at 10 °C was only 60% its initial value, while it was 82.5% for the membrane prepared at 50 °C. This is because the membrane cross-sectional morphology when prepared at low temperatures was more open with large finger-like cavities inside, which makes it more susceptible to compression by pressure. Indeed, a comparison of the compression effect for the membrane prepared at 25 °C shows that, when being operated at 0.25 bar, it sustained almost the same flux, while a decrease of about 25% was recognized when it was operated at 1.25 bar (Figure 11). However, this effect was somewhat reversible, as the membrane recovered part of its permeability after being immersed in water for 24 h after the first test, the flux decrease appeared again in the second test with even a higher rate. Given this trade-off, the selection of preparation temperature is quite important in determining the desired properties and performance of the membranes.

## 4. Conclusions

In this study, neat PVDF and composite PVDF-TiO_2_ membranes were prepared at four different temperatures (10, 25, 50 and 70 °C) by the NIPS method. The temperature was closely controlled and synchronized in each step of the procedure to avoid the influence of thermal gradient. The results showed that preparation temperature significantly influenced the membrane formation process, as well as the final membrane properties.

It appeared that, with increasing temperature, the polymer solutions (for both PVDF and PVDF-TiO_2_) were more thermodynamically stable, which means it was more difficult to disturb them to induce phase separation. On the contrary, the phase separation kinetics were stronger at higher temperatures, i.e., the solvent–nonsolvent exchange rate was faster. This trade-off makes it difficult to explain the evolution in membrane morphology with temperature, as seen in the SEM images. On the other hand, the direct microscopic observation of the membrane formation during phase separation, especially at the entrance of water, when in contact with the polymer solutions, revealed a stark contrast between the processes at low and high temperatures. This partly explains the evolution in temperature of the finger-like macrovoids in the membrane cross section, i.e., macrovoids diminished in size and amount upon the increase in membrane preparation temperature, as observed by SEM.

The SEM results also revealed an increase in the surface pore size and frequency with temperature, while also showed systematic agreements with other membrane properties such as porosity, permeability, mechanical strength and compression behavior. In particular, the permeability decreased with the membrane preparation temperature, suggesting it is strongly related to the presence of the finger-like macrovoids in membrane structure. A trade-off between membrane permeability and mechanical strength was identified. In addition, the thermal behavior of membranes and their crystalline properties also showed systematic transitions when the temperature increased. Therefore, it is suggested as a rule that the membranes can be adjusted to be more robust with an increase in preparation temperature, while for them to be more permeable, the temperature should be reduced. In general, this study is expected to provide a systematic approach to characterizing the PVDF-TiO_2_ membranes and contributing to understanding some existing inconsistencies regarding this type of composite membrane in the literature.

It should also be noted that, as expected, the composite PVDF-TiO_2_ membranes showed a superior permeability compared to the neat PVDF membranes (for example, almost 10 times higher when both were prepared at 25 °C), due to the addition of TiO_2_. The superior photo-induced hydrophilicity of PVDF-TiO_2_ membranes was already demonstrated in our previous study [31]. The only drawback of these membranes is their reduced mechanical strength compared to the neat PVDF membranes, which can be explained by the finger-like structures and their crystalline properties. Other membrane performance indicators, such as separation and photocatalytic filtration, are subjected to be investigated in future studies.

## Figures and Tables

**Table 1:** Proposed mechanisms of macrovoids formation in the literature.

Mechanism	References
**Shrinkage of polymer matrix** Initiation of macrovoids is induced by fractures in skin layer of the polymer solution, then the shrinkage of polymer matrix drains new precipitates to the finger side, enlarging the macrovoids. Fast polymer precipitation rate often leads to more macrovoids structures.	[42,43]
**Surface tension gradient-induced convective flow** Interfacial tension of the polymer solution/water interface becomes zero at one point, leading to water intrusion in the polymer solution and the initiation of macrovoids. Afterwards, solvent diffusion to the intrusions cause the macrovoids to grow in size. Processes occurring at ambient temperature often lead to finger-like structures. Preliminary evaporation time before immersion leads to decreases in size and number of macrovoids.	[41,44,45,46,47]
**Instantaneous phase demixing** Initiation of macrovoids is induced by the expansion of nuclei droplets of the polymer-lean phase to very large dimensions, then the combined effect of diffusional flows of the polymer lean phase, relative to the polymer-rich phase and liquid–liquid demixing, leads to the growth of macrovoids. Instantaneous demixing leads to more finger-like macrovoids.	[46,48,49]
**Viscous fingering** Osmotic pressure leads to water diffusion into polymer-lean phase and causes the initiation of macrovoids. Then, viscous fingering causes the forming of complex finger-like patterns of water inside the polymer solution, leading to macrovoids growth. In addition, delayed demixing also occurs at the walls of the fingers, leading to the propagation of the phase separation front. If the viscous fingering rate is faster than the phase separation front, finger-like structures are more favored.	[50,51]

**Table 2:** Melting temperature (T_m_), total crystallinity (X_c_) and fraction of α crystalline form (F_α_) of membranes (the number after membrane name indicates the temperature (°C) at which it was prepared).

Membrane	T_m_ (°C)	X_c_ (%)	F_α_ (%)	Membrane	T_m_ (°C)	X_c_ (%)	F_α_ (%)
PVDF-10	165.9 ± 0.0	61.6 ± 1.0	38.9%	PVDF-TiO_2_-10	164.5 ± 0.2	61.2 ± 1.3	36.4%
PVDF-25	166.7 ± 0.2	58.0 ± 1.2	56.6%	PVDF-TiO_2_-25	164.7 ± 0.1	60.3 ± 1.2	42.7%
PVDF-50	167.4 ± 0.1	59.0 ± 0.7	64.2%	PVDF-TiO_2_-50	165.4 ± 0.1	58.4 ± 0.8	55.9%
PVDF-70	167.5 ± 0.2	54.8 ± 0.7	68.8%	PVDF-TiO_2_-70	165.9 ± 0.0	55.8 ± 2.1	60.3%
